# Osteoporosis Screening Using Dental Panoramic Radiographs and Age at Menarche

**DOI:** 10.3390/diagnostics13050881

**Published:** 2023-02-24

**Authors:** George Triantafyllopoulos, Anastasia Mitsea, Aliki Rontogianni, Demitrios Korres

**Affiliations:** 1 Medical School, National and Kapodistrian University of Athens, 11527 Athens, Greece; 2 Dental School, National and Kapodistrian University of Athens, 11527 Athens, Greece

**Keywords:** osteoporosis, panoramic radiographs, DXA examination, radiomorphometric indices, clinical risk assessment tools, age at menarche

## Abstract

Since early detection of osteoporosis is essential, the development of an efficient and cost-effective screening model would be incredibly beneficial. The aim of this study was to evaluate the diagnostic accuracy of MCW and MCI indices from dental panoramic radiographs in combination with a new variable, age at menarche, for the detection of osteoporosis. The study enrolled 150 Caucasian women (aged 45 to 86) who met the eligibility criteria, had DXA scans of the left hip and lumbar spine (L2 to L4), and were classified as osteoporotic, osteopenic, or normal based on T-score. Two observers evaluated MCW and MCI indexes on panoramic radiographs. There was a statistically significant correlation between the T-score and MCI and MCW. In addition, age at menarche had a statistically significant correlation with T-score (*p* = 0.006). In conclusion, in the current study, MCW proved to be more effective in detecting osteoporosis when combined with age at menarche. Individuals with MCW less than 3.0 mm and age at menarche later than 14 years old should be referred for DXA since they present high risk of osteoporosis.

## 1. Introduction

Osteoporosis primary diagnosis is advantageous for both patients’ quality of life and the public healthcare system by reducing overall healthcare expenses. Therefore, developing an osteoporosis screening model that is effective, inexpensive, and simple to use is essential. Accordingly, several efforts have been made in recent decades to develop alternative, low-cost screening tools to detect females at high risk of osteoporosis [1].

A substantial number of dental radiographs are performed every day for dental therapeutic purposes. So, the researchers sought to obtain data from dentomaxillofacial images, which could contribute to the early diagnosis of osteoporosis. As has emerged from relevant studies, qualitative and quantitative evaluations in panoramic images may contribute to osteoporosis primary diagnosis. These evaluations were performed with the application of radiomorphometric indices. After expanded research, it has emerged that the three most noteworthy radiomorphometric indices are the mandibular cortical width (MCW), the panoramic mandibular index (PMI), and the mandibular cortical index (MCI) [2,3,4,5,6,7].

MCW is defined in panoramic radiographs as the width of the inferior cortex of the mandible below the mental foramen. This index has been suggested to be a good predictor of skeletal bone mineral status and seems to be a useful tool for assessing a patient’s risk of osteoporosis [8,9]. Despite its widespread use, there is no agreement on the ideal cortical thickness cut-off point for referral for bone densitometry. An MCW cutoff point of 3 mm or less has been suggested, below which referral would be recommended [2,3,4,8,9].

Furthermore, the MCI index is a three-stage qualitative evaluation of the mandible’s lower border porosity that is connected to bone mineral density and which has already been confirmed to be a suitable technique for osteoporosis screening [2,3,10,11,12,13,14,15,16,17]. MCI is applied bilaterally on panoramic radiographs at the lower border of the mandible posterior to the mental foramen and is classified subjectively into three stages. In stage 1, the cortical endosteal margin looks even and consistent. When the endosteal margin presents signs of semilunar deficiencies or one to three layers of cortical endosteal remain, it is categorized as stage 2. The cortical layer reaches stage 3 when it has numerous (>3) endosteal semilunar deficiencies and is obviously porous. The higher category (stage 3) indicates a significantly higher likelihood of osteoporosis compared to the lower (stage 2). Since MCI usually presents good sensitivity but low specificity, which can result in increased false positive results and needless DXA examinations, it is preferable to be used in combination with other indexes or clinical risk assessment tools [2,3,4,5,6,7,11].

In addition to the above radiographic indexes, there are several clinical risk assessment tools that take into consideration multiple risk parameters for osteoporosis and are used to identify people who could benefit from DXA advice [18,19,20,21]. MCW was found to have comparable sensitivity and specificity to one clinical risk evaluation approach, OST (Osteoporosis Self-Assessment Tool), but lower diagnostic validity than OSIRIS (Osteoporosis Index of Risk) [8,9,22,23]. To enhance the diagnostic validity of the aforementioned radiomorphometric indices, an additional variable should be introduced.

The aim of the current study was to evaluate the diagnostic validity of the MCW and MCI indices for screening osteoporosis in women when applied to dental panoramic radiographs in combination with a new variable, age at menarche.

## 2. Materials and Methods

### 2.1. Study Population

Over a twelve-month period, 150 Caucasian women, with ages ranging from 45 to 86 years (mean age 61.79 years), who attended a public general hospital’s dental department seeking dental treatment, were enrolled in the study (Table 1). The research was carried out in compliance with the Helsinki Declaration (WMA 2013), ethical approval was obtained, and all participating women provided informed consent before being enrolled in the study [24]. The exclusion criteria included age under 45 years, secondary osteoporosis, primary hyperparathyroidism, poorly controlled thyrotoxicosis, malabsorption syndrome, liver disease, alcohol addiction, and unsatisfactory dental panoramic images. Each subject’s age, body height, body weight, menopausal status, and age at menarche were also registered.

### 2.2. Bone Mineral Density Measurements

To establish the gold standard for the study, dual-energy X-ray absorptiometry scans (DXA scans) of the left hip and lumbar spine (L2 to L4) were undertaken on every individual using a General Electric Lunar Prodigy KO. Individuals with a T-score value of −2.5 standard deviations (SD) or more below the young female adult mean bone mineral density value, at any of total hip, femoral neck, or lumbar spine, were categorized as osteoporotic according to WHO requirements for Caucasian women. Subjects with T-scores ranging from −1 to −2.5 SD were characterized as osteopenic, whereas the rest were categorized as normal [25].

### 2.3. Radiographic Measurements

As part of the patient’s treatment plan, dental panoramic radiographs were obtained with a General Electric Orthopantomograph^®^ OP100. The imaging parameters differed according to the subject’s anatomy, but were typically 70 kV at 8 mA for 15 s. When the dental panoramic radiograph was taken, all subjects held a plastic bite block containing a 3.175 mm diameter metal ball between their left premolar teeth. To correct the magnification errors of the linear measurements, the predefined dimensions of the metallic ball were used as a reference. All measurements were conducted on radiographic films.

**Figure 1:**
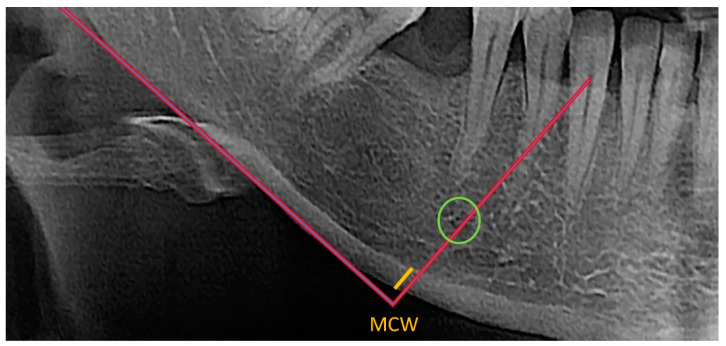
Evaluation of the thickness (yellow line) of the cortical bone at the lower border of the mandible (MCW index) below and distally of the mental foramen (green circle).

MCW was assessed by two independent observers who used the OSTEODENT project methodology on dental panoramic radiographs of both sides of the mandible. As shown in Figure 1, a line was traced from every foramen’s midpoint to the mandible’s lower border, which was then tangential to the lower border. Along this line, the thickness of the cortical bone at the lower border was evaluated [22,26,27]. All measurements were conducted using ImageJ software and recorded down to the closest 0.1 mm. Each patient’s bilateral measurements were averaged. The mean diameter of the metal ball was calculated using the measured width and height of the ball-bearing image [22].

Additionally, applying the methodology described by Klemetti et al., the same observers blindly and independently classified the appearance of the endosteal edge of the mandibular cortical bone (MCI) as seen posterior to the mental foramen on both sides of each individual’s dental panoramic radiographs [10].

**Figure 2:**
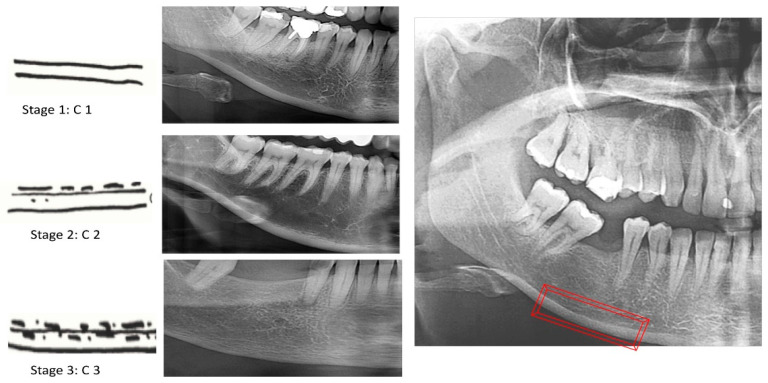
Classification of the MCI index (The area of evaluation in red).

MCI is a three-stage index, as shown in Figure 2:

C1: the endosteal edge of the cortex is even and precise on both sides.

C2: the endosteal edge exhibits semilunar deficiencies (lacunar resorption) or appears to have endosteal cortical fragments (1–3 layers), solely on a single side or on both sides.

C3: the cortex is obviously porous and has intense endosteal cortical residue fragments.

The observers were uninformed of the subjects’ reference standard BMD results obtained. All measurements were repeated independently by both observers after an interval of one month.

### 2.4. Statistical Analysis

Data concerning the quantitative variables were presented as the mean and standard deviation (SD) and as percentages for categorical variables. The Kolmogorov–Smirnov test was applied to evaluate the normality of the quantitative variables. When data normality was violated, logarithmic transformation and non-parametric analysis were applied.

Interobserver agreement for the MCI and MCW indices was estimated with the linear weighted kappa and interclass correlation coefficient (ICC).

Bivariate analyses were performed using the Student’s *t*-test. The relationships between the outcome variable (MCW-T-score-age at menarche) and the quantitative variables, were analyzed using one-way ANOVA. The Pearson correlation coefficient was used to analyze the relationships between the outcome variable and the qualitative measures. 

Univariate analyses were performed using the chi-square test, or, alternatively, the Fisher exact test was employed to investigate the relationship between the outcome variable (osteoporosis, yes or no) and the qualitative variables, whereas the Student’s t-test or Mann–Whitney U-test and one-way ANOVA or Kruskal–Wallis tests were employed to investigate the connection between the outcome measure and the quantitative variables. The Cohen’s kappa was used according to Landis and Koch standards to assess the agreement between the DXA T-score and MCI as follows: bad (k < 0), slight (0 < k < 0.2), fair (0.21 < k < 0.40), moderate (0.41 < k < 0.60), significant (0.61 < k < 0.80), and excellent (0.81 < k < 1.00).

By calculating the respective areas under the curve (AUC), a receiver operating curve (ROC) analysis was performed to assess the diagnostic performance and to obtain cut-off levels of the MCW index for the categorization of patients as osteoporotic and non-osteoporotic. The AUC areas, with their standard error and 95% MCI, were estimated using the maximum likelihood estimation technique, which has the benefit of not requiring hypotheses about the Gaussian distribution of the underlying variables.

All demographic, bone, and dental panoramic radiography markers, whether or not they demonstrated significant associations with outcome variables (osteoporosis vs. no osteoporosis) in univariate analysis, were included in the multiple logistic regression model. To evaluate the quality of fit, the Hosmer–Lemeshow test was applied. The sample data were indicative of the general population’s data. All tests were two-sided, and statistical significance was denoted by a *p*-value of 0.05. The statistical program SPSS version 17 was used for all analyses (Statistical Package for the Social Sciences, SPSS Inc, Chicago, IL, USA).

## 3. Results

The study group’s average age was 61.79 years (SD ± 9.16) with individuals’ ages ranging from 45 to 86 years. The average age at menarche was 13.12 years (SD ± 1.52), with a range of 9 to 17 years. Table 1 provides additional descriptive statistics for the sample. The average age at menarche has decreased in recent decades to 12–13 years of age [28]. According to the age range of the sample, the individuals were likely born several decades ago, so we set a cut-off point of 14 years of age at menarche for the statistical analysis of the sample data.

Osteoporotic individuals (T-score < 2.5), with osteoporosis either in the lumbar spine or in the femoral neck, were found with a statistically significant higher age at menarche (*p* = 0.034 and 0.035, respectively) when compared with normal subjects, as seen in Table 2.

Table 3 presents the agreement between the two observers for the MCI index, which was found to be excellent (Cohen’s kappa index 0.831). Interobserver agreement concerning the MCW index was found to be excellent (ICC 0.8)

As seen in Table 4, between the lumbar spine T-score and the MCI, there was a statistically significant correlation (*p* = 0.001). When comparing the femoral neck T-score and MCI, a comparable statistically significant correlation was observed (*p* = 0.009).

A strong correlation between MCW and the lumbar spine T-score (*p* = 0.001), as well as the trochanteric neck T-score (*p* < 0.0005), was found, as presented in Table 5.

Table 6 presents the relationship between the three categories of T-score (normal, osteopenic, and osteoporotic individuals for the lumbar spine and femoral neck) and the MCW measurements, which were found to be statistically correlated (*p* < 0.0005).

**Figure 3:**
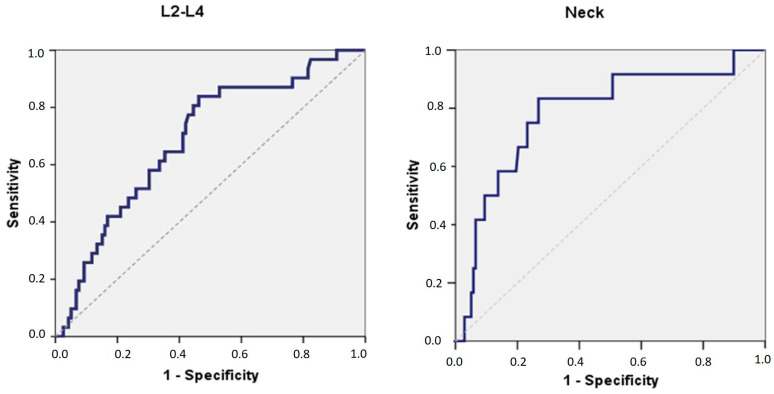
Receiver operator characteristic (ROC) curves of mandibular cortical width (MCW) with T-score for the lumbar spine(L2–L4) and the femoral neck, respectively.

ROC analysis showed that, in the lumbar spine, the area under the curve (AUC) for MCW was 0.689 (95% MCI 0.59–0.79 *p* = 0.001), with a cut-off point of 3.42, 84% sensitivity, and 54% specificity. Concerning the femoral neck, the AUC for MCW was 0.783 (95% MCI 0.64–0.93 *p* = 0.001), with a cut-off point of 3.00, 75% sensitivity, and 77% specificity. As seen in Table 7 and Figure 3, for both the lumbar spine and trochanteric neck, the AUC for MCW was 0.885 (95% MCI 0.79–0.98 *p* = 0.009), with a cut-off point of 3.00, 100% sensitivity and 75% specificity.

If an individual’s age at menarche was above 14 years old, she had 80% less probability of developing osteoporosis, and if MCW was less than 3.4, the individual had 3.9 times greater probability of developing osteoporosis.

## 4. Discussion

While bone loss starts earlier in a woman’s life, after menopause, oestrogen insufficiency impairs bone tissue turnover and promotes skeletal bone degeneration. One of the most severe medical conditions affecting post-menopausal women is osteoporosis, which affects bone microarchitecture. The most significant issue that osteoporotic patients encounter is an increasing incidence of fractures. Since the implications range from effects on the patients’ quality of life to economic effects on the healthcare system, asymptomatic individuals must be detected as early as possible. In order to detect osteoporotic patients at risk of fracture in the next ten years, the WHO introduced FRAX, a diagnostic tool that predicts the possibility of a patient (older than 50 years of age) experiencing a fracture, based on 11 clinical factors other than BMD [8,11,17,22,27,29,30,31,32,33,34,35,36].

Concerning the jaws, both trabecular and cortical bone are deteriorated in osteoporotic individuals. Bone resorption mostly occurs in the nutrient canals of the cortical bone. The surrounding canals progressively converge, causing the cortical bone to thin. Thinning of the lower cortex of the lower jaw is visible radiographically [35,37]. Consequently, careful evaluation of dental radiographs can provide the clinician with valuable information concerning the screening of osteoporotic patients. Many reports exist concerning the usefulness of oral radiographs, and especially panoramic radiographs, in osteoporosis early diagnosis [8,11,17,22,27,29,30,31,32,33,34,35].

In particular, the evaluation of MCW and MCI radiomorphometric indices in panoramic radiographs has been proven to be a useful screening tool for individuals at risk of developing osteoporosis. Although this method seems efficient and cost-effective, there are studies showing that moderate to good sensitivity is limited by low specificity. A more reliable screening tool could be developed if we managed to increase both the sensitivity and specificity. To overcome the above problem, it is crucial to identify an accurate diagnostic threshold [3,4,5,6,7,11,22,30].

According to the results of the present study, MCI was less accurate in detecting osteoporosis than MCW. This finding is in agreement with the findings of other studies [1,13,33,38,39,40,41]. However, as indicated by the findings of other studies, the MCI score is a beneficial diagnostic tool for the identification of osteoporotic individuals [35]. All the aforementioned different findings about the MCI’s ability to detect osteoporotic individuals are illustrated by the range of the MCI’s sensitivity in detecting osteoporosis, which varies from 35.9 to 90.9% [36]. Therefore, it should be noted that the evaluation of radiographs for the application of the MCI index should be carried out with extreme caution, since rapid examination of radiographs might result in incorrect results. In particular, a thoughtless evaluation of the cortical bone margin may result in a misdiagnosis of cortical erosion even though the bone is normal since the trabecular bone can appear to be attached to the cortical bone. Moreover, bone erosion might be considered as normal [26].

On the other hand, MCW seems to be more effective at classifying subjects who are at risk of developing osteoporosis, which is consistent with the results of earlier studies [6,11,30,31,32,33,34]. In particular, MCW seems more effective than other radiomorphometric indices, but is not entirely satisfactory since it has a low capacity for differentiating subjects into the three categories (normal, osteopenic, osteoporotic) [34,35,36].

Earlier studies reported a statistically significant correlation between MCW and T-score [16,19,20]. Devlin and Horner reported that the MCW index was significantly correlated to the jaw BMD, whereas in the OSTEODENT study, a significant correlation was also reported between MCW and BMD in the jaw and in the femoral neck [4,22,42].

According to the results of this study, receiver operating characteristic (ROC) curve analysis showed that, in the lumbar spine, the area under the ROC curve (AUC) for MCW was 0.689 (95% MCI 0.59–0.79 *p* = 0.001), with a cut-off point of 3.42, 84% sensitivity and 54% specificity. This indicates that, with respect to the sample of Caucasian women in this study, the MCW index is moderately accurate. Regarding the femoral neck, the AUC for MCW was 0.783 (95% CI 0.64–0.93 *p* = 0.001), with a cut-off point of 3.00, 75% sensitivity, and 77% specificity. Similarly, this indicates moderate accuracy for the MCW index. When both the lumbar spine and femoral neck were evaluated, the AUC for MCW was 0.885 (95% MCI 0.79–0.98 *p* = 0.009), with a cut-off point of 3.00, 100% sensitivity, and 75% specificity). This indicates that the accuracy of the MCW index seems to be high. These results are similar to those obtained in studies performed on other population samples [34].

Several worldwide research studies have attempted to correlate the MCW value to the identification of individuals at risk of osteoporosis. Numerous cut-off values have been documented in the literature in an attempt to define the ideal threshold for the use of MCW values as a trustworthy predictive index for low BMD. In a study concerning a Korean post-menopausal population, a lower cortical thickness threshold of 2.2 mm was observed. This study suggested that, if a patient’s mandibular cortical thickness is less than 2.5 mm, they should be referred for further osteoporosis evaluation. In the Japanese population, a cortical thickness threshold of 2.8 mm has been found. In both European and Brazilian populations, a 3.0 mm threshold has been found. An upper threshold of 4.5 mm has been found in a Saudi Arabian population. The threshold of 4 mm has been shown to be inadequate for categorizing women into normal, osteopenic, and osteoporotic categories. In accordance with the published research data, a 3 mm cutoff point is an appropriate cutoff point for screening patients at risk of osteoporosis [1,4,10,12,22,34,35,42,43,44].

A cut-off point of 3.0 mm of MCW was established in the current study as the threshold for referral for DXA evaluation, which showed 100% sensitivity and 75% specificity for both the lumbar spine and femoral neck. According to previous studies, osteoporosis occurrence and the timeframe since menopause are related [45].

Since there are limited data relating the age of menarche to the risk of osteoporosis, we thought it would be beneficial to investigate the relationship between the age at menarche, the MCW index, and the risk of osteoporosis [46]. When used in practice, a combined measurement that takes into account both MCW and age at menarche above 14 years old results in an 80% lower likelihood of developing osteoporosis. Therefore, it is reasonable to suggest that an MCW threshold of 3 mm, combined with age at menarche, would be more accurate.

The outcomes of the numerous types of research that have been conducted concerning the usefulness of oral imaging examinations in osteoporotic patients screening are variable [2,3,12,22,39,40,41]. The varied results may be related to different study designs, observer experience, ethnic or racial variances, or to different statistical analyses [34,42,43]. Based on all the relevant studies, it is unquestionable that appropriate dentist knowledge and experience for evaluating patients’ dental radiographs and deciding on further referral, may contribute to the early diagnosis of osteoporosis [12,22,35,36,37,38,39,40,41,42,43,44].

Within the framework of continuing education and professional development, dentists should be informed about the possibility of screening osteoporotic patients by panoramic radiographs and, particularly, by means of radiomorphometric indices. They should be trained not only in the application of radiomorphometric indices, but also in the evaluation and synthesis of data from the patient’s history, such as the age at menarche [35].

Recently, several efforts have been made to use computer-aided diagnosis systems for the diagnosis of osteoporosis. The objective has been to create a reliable, unbiased, simple, and affordable method for osteoporosis diagnosis. The outcomes have been encouraging, and the prospects for future research are promising. Regardless of these advancements, the use of dental radiographs to economically, quickly and easily detect undiagnosed osteoporotic patients will always be essential [35,36,47,48,49,50,51].

## 5. Conclusions

In conclusion, the present study demonstrated that MCW is more efficient in detecting osteoporosis when it is combined with age at menarche. If an individual’s age at menarche is above 14 years old, she has an 80% less probability to develop osteoporosis, and if MCW is less than 3.4 mm, the individual has a 3.9 times greater probability to develop osteoporosis.

Consequently, individuals with an MCW score less than 3.0 mm and age at menarche above 14 years old should be referred for DXA as the likelihood of osteoporosis is significant.

## Figures and Tables

**Table 1:** Sample descriptives.

Demographic Data	Mean	SD	Minimum	Maximum
Age (yrs)	61.79	9.16	45.00	86.00
Height (cm)	157.93	6.28	144.00	173.00
Weight (kgr)	76.79	14.90	45.00	134.00
BMI	30.85	6.15	18.73	54.36
Age at menarche	13.12	1.52	9.00	17.00
Menopause (yrs)	49.59	3.54	45.00	60.00
Years from menopause	12.23	9.16	0.00	41.00

**Table 2:** Correlation between T-score measurements and age at menarche.

		Ν	Mean Age at Menarche	SD	*p*-Value
T-score L2–L4	normal	89	12.89	1.27	0.040
	osteopenic	30	13.23	1.74	
	osteoporotic	31	13.68 *	1.83	
T-score Neck	normal	90	12.92	1.37	0.035
	osteopenic	48	13.25	1.65	
	osteoporotic	12	14.08 *	1.78	

* *p* < 0.05 vs. normal.

**Table 3:** Interobserver reliability for MCI index.

MCI 1st Observer									
**MCI 2nd observer**		**C1**		**C2**		**C3**		**Total**	
		**N**	**%**	**N**	**%**	**N**	**%**	**N**	**%**
	**C1**	**66**	**44.0**	0	0	0	0	66	44.0
	**C2**	10	6.7	**55**	**36.7**	0	0	65	43.3
	**C3**	0	0	5	3.3	**14**	**9.3**	19	12.7
	**Total**	76	50.7	60	40.0	14	9.3	150	100.0

**Table 4:** Comparison of T-score at different areas among MCI categories.

		Ν	Mean	SD	*p*-Value
T-score L2-L4	C1	66	−0.79 *	1.44	0.001
	C2	65	−0.98 *	1.63	
	C3	19	−2.24	1.26	
T-score Neck	C1	66	−0.84 *	1.10	0.009
	C2	65	−1.11 *	1.11	
	C3	19	−1.71	0.85	
T-score Total	C1	66	−0.49 *	1.10	0.036
	**C2**	65	−0.55 *	1.14	
	**C3**	19	−1.22	1.03	

* *p* < 0.05 vs. C3.

**Table 5:** Correlation between T-score and MCW.

	ICC	95% CI	*p*-Value
**T-score L2–L4**	0.406	0.18–0.57	0.001
**T-score Neck**	0.515	0.33–0.65	<0.0005

**Table 6:** Comparison of MCW score among T-score categories at different areas.

		N	MCW Mean	SD	*p*-Value
**T-score L2-L4**	normal	89	3.65	0.66	<0.0005
	osteopenic	30	3.09 *	0.67	
	osteoporotic	31	3.11 *	0.55	
**T-score Neck**	normal	90	3.61	0.67	<0.0005
	osteopenic	48	3.21 *	0.64	
	osteoporotic	12	2.88 *	0.56	

* *p* < 0.05 vs. normal.

**Table 7:** ROC analysis of MCW with T-score in the lumbar spine and femoral neck.

	AUC	SE	*p*-Value	Cut-Off Point	Sensitivity	Specificity	95% C.I.	
L2-L4	0.689	0.051	0.001	3.42	84%	54%	0.588	0.789
Neck	0.783	0.073	0.001	3.00	75%	77%	0.640	0.926
Total	0.885	0.048	0.009	3.00	100%	75%	0.792	0.979

AUC: Area under the curve.

## Data Availability

Data can be made available upon reasonable request.
